# Understanding Resistance vs. Susceptibility in Visceral Leishmaniasis Using Mouse Models of *Leishmania infantum* Infection

**DOI:** 10.3389/fcimb.2019.00030

**Published:** 2019-03-01

**Authors:** Begoña Pérez-Cabezas, Pedro Cecílio, Tiago Bordeira Gaspar, Fátima Gärtner, Rita Vasconcellos, Anabela Cordeiro-da-Silva

**Affiliations:** ^1^Instituto de Investigação e Inovação em Saúde, Universidade do Porto, Porto, Portugal; ^2^Parasite Disease Group, Instituto de Biologia Molecular e Celular, Universidade do Porto, Porto, Portugal; ^3^Departamento de Ciências Biológicas, Faculdade de Farmácia, Universidade do Porto, Porto, Portugal; ^4^Cancer Signalling and Metabolism Group, Institute of Molecular Pathology and Immunology of University of Porto, Porto, Portugal; ^5^Faculdade de Medicina, Universidade do Porto, Porto, Portugal; ^6^Instituto de Ciências Biomédicas Abel Salazar, Universidade do Porto, Porto, Portugal; ^7^Department of Molecular Pathology and Immunology, Instituto de Ciências Biomédicas Abel Salazar, Universidade do Porto, Porto, Portugal; ^8^Glycobiology in Cancer Group, Institute of Molecular Pathology and Immunology of University of Porto, Universidade do Porto, Porto, Portugal; ^9^Immunobiology Department, Biology Institute, Universidade Federal Fluminense, Rio de Janeiro, Brazil

**Keywords:** *Leishmania*, visceral leishmaniasis, mouse models, susceptibility vs. resistance, immune regulation

## Abstract

Every year, up to 90,000 new cases of Visceral Leishmaniasis and 30,000 resultant deaths are estimated to occur worldwide. Such numbers give relevance to the continuous study of this complex form of the disease: a zoonosis and an anthroponosis; two known etiological agents (*Leishmania infantum* and *L. donovani*, respectively); with an estimated average ratio of 1 symptomatic per 10 asymptomatic individuals; and sometimes associated with atypical clinical presentations. This complexity, which results from a long co-evolutionary process involving vector-host, host-pathogen, and pathogen-vector interactions, is still not completely understood. The determinants of visceralization are not fully defined and the dichotomy resistance vs. susceptibility remains unsolved, translating into obstacles that delay the progress of global disease control. Inbred mouse models, with different susceptibility patterns to *Leishmania* infection, have been very useful in exploring this dichotomy. BALB/c and C57BL/6 mice were described as susceptible strains to *L. donovani* visceral infection, while SV/129 was considered resistant. Here, we used these three mouse models, but in the context of *L. infantum* infection, the other *Leishmania* species that cause visceral disease in humans, and dynamically compared their local and systemic infection-induced immune responses in order to establish a parallel and to ultimately better understand susceptibility vs. resistance in visceral leishmaniasis. Overall, our results suggest that C57BL/6 mice develop an intermediate “infection-phenotype” in comparison to BALB/c and SV/129 mouse strains, considering both the splenic parasite burden and the determined target organs weights. However, the immune mechanisms associated with the control of infection seem to be different in each mouse strain. We observed that both BALB/c and SV/129, but not C57BL/6 mice, show an infection-induced increase of splenic T follicular helper cells. On the other hand, differences detected in terms of CD21 expression by B cells early after infection, together with the quantified anti-*Leishmania* specific antibodies, suggest that SV/129 are faster than BALB/c and C57BL/6 mice in the assembly of an efficient B-cell response. Additionally, we observed an infection-induced increase in polyfunctional CD4+ T cells in the resistant SV/129 model, opposing an infection-induced increase in CD4+IL-10+ cells in susceptible BALB/c mice. Our data aligns with the observations reported for *L. donovani* infection and suggest that not only a single mechanism, but an interaction of several could be necessary for the control of this parasitic disease.

## Introduction

More than a century after the discovery of leishmaniasis and its vector-borne causative agent, *Leishmania* spp., a lot of ground remains to be covered. The number of species described associated with human disease has been increasing [around 20 species with clinical relevance (Akhoundi et al., 2016)] and, with them, the complexity of the host-parasite interactions equation. It is well-accepted that the infection outcome depends on a number of factors including the infecting parasite species, and the “equilibrium” between the host immune response and the parasite immune-evasion strategies (Cecílio et al., 2014). These aspects justify the different known leishmaniasis clinical manifestations (that vary from a localized cutaneous ulcer to skin and mucosa metastatic lesions, or to the colonization of internal organs such as the spleen, liver, and bone marrow), consequently associated with different pathological mechanisms (Bates, 2007; Hartley et al., 2014). Every year up to one million new cases and 30,000 deaths are associated with this spectrum of diseases (World Health Organization, 2017).

The quest for the missing vaccine and for better therapeutic options for human leishmaniasis requires the understanding of the infectious process (from the transmission of *Leishmania* parasites by their phlebotomine vector) which is still not completely understood. The determinants of metastization (diffuse cutaneous leishmaniasis; mucocutaneous leishmaniasis; PKDL) and visceralization (visceral leishmaniasis) are still ambiguous, while the susceptibility vs. resistance dichotomy remains unestablished for some disease forms (McCall et al., 2013; Hartley et al., 2014). The use of murine inbred animal models was indispensable for the establishment of the Th1/Th2 paradigm which explains resistance vs. susceptibility (respectively) to cutaneous disease (Sacks and Noben-Trauth, 2002), and for the disclosure of genetic resistance determinants in visceral disease, such as the expression of Nramp1 antiporter, that when functional, prevents parasite replication in the phagolysosome, by limiting their access to essential divalent cations (Lipoldova and Demant, 2006; Kumar and Nylén, 2012). Still, in visceral disease, the immunological aspects that condition parasite persistence and their connection with host genetic factors needs to be further explored, in a way to definitively understand resistance vs. susceptibility.

Here, taking advantage of three inbred mouse strains, with known different susceptibility patterns to infection by the viscerotropic *L. donovani* species (Lipoldova and Demant, 2006), we compared the development of experimental *L. infantum* infection, the main causative agent of visceral leishmaniasis in South America and the Mediterranean Basin (Ready, 2014). For this, at two different time-points, we quantified the parasite burdens in the main target organs; evaluated the liver's granulomatous responses; studied the splenic immune cell compartment composition and their infection-induced modifications, particularly emphasizing T and B lymphocyte's phenotypes; and assessed the development of specific humoral responses against the parasite, as a way to explore the above-mentioned dichotomy. The data obtained complement findings recently reported for *L. donovani* infection (Bodhale et al., 2018), important for the establishment of a parallel between the viscerotropic *Leishmania* strains in the context of *in vivo* infections.

## Materials and Methods

### *L. infantum* Culture

A cloned line of virulent *Leishmania infantum* (MHOM/MA/67/ITMAP-263) freshly recovered from BALB/c mice was used for a total of 10 passages. Promastigotes were routinely maintained at 26°C in standard RPMI 1640 medium supplemented with 10% heat-inactivated Fetal Calf Serum (FCS; Biowest, Nuaillé, France), 2 mM L-glutamine, 100 U/ml penicillin, 100 μg/ml streptomycin and 20 mM HEPES buffer, all products from Lonza (Basel, Switzerland). All maintenance cultures were grown with a starting inoculum of 10^6^ parasites/ml. Parasites for *in vivo* infections were always collected after 5 days of culture.

### Mouse Strains, Infections, and Euthanasia

Six- to eight-week-old sex-matched BALB/c, C57BL/6 and SV/129 mice (Charles River Laboratories, France) were maintained under specific pathogen-free conditions at the i3S facilities, in sterile IVC cabinets, with food and water available *ad libitum*. The three mouse strains were always infected in parallel with the same parasite's preparation. Each animal was infected intraperitoneally with 1 × 10^8^ stationary promastigotes, prepared as reported elsewhere (Faria et al., 2016). Two or eight weeks after infection, mice were anesthetized with isoflurane (Piramal healthcare, Northumberland, UK) and further manipulated only after the total loss of pedal reflex (firm toe pinch). Euthanasia was performed by cervical dislocation (under volatile anesthesia). All the controls (non-infected) used in the experiments were strain-, age-, and sex-matched.

### Blood and Organ Collection and Manipulation

Blood from mice was collected through intracardiac puncture under isoflurane anesthesia. Serum was collected and stored at −80°C for posterior analysis. Spleens and livers were aseptically collected from euthanized animals, weighed, and either homogenized using Falcon®100 μm Cell Strainers (Corning Life Sciences, Tewksbury, MA, USA) and manual Potter-Elvehjem tissue homogenizers, respectively, or preserved in formalin for posterior histological evaluation.

### Determination of Parasite Burdens

Splenic and hepatic parasite burdens were assessed using the limit dilution method, starting from 1 and 5 mg of organ, respectively. The “parasite titer” was considered the last dilution with >1 motile parasite. The number of parasites per gram of organ was calculated as previously described (Silvestre et al., 2007).

### Histopathology

Livers were fixed in 10% formaldehyde (pH 7.4) for 48 h, followed by dehydration in ethanol and clarification in xylene (all from Sigma-Aldrich, MO, USA). Tissues were embedded in paraffin and cut to a thickness of 4 μm. All sections were stained with Hematoxylin and Eosin (H&E) for histopathological analysis. Slides were observed in an Axioskop 2 Zeiss microscope (Carl Zeiss, Jena, Germany) and photographs (100 and 400X magnifications) were acquired using a Nikon DS-L1 camera (Nikon, Tokyo, Japan). Two distinct observers blindly evaluated the preparations. The total number of granulomas was determined for each animal by accounting 20 microscopic fields (100x magnification). In cases with a very poor granulomatous response, the total number of granulomas *per* slide was accounted for.

### Flow Cytometry

The anti-mouse monoclonal antibodies used to perform this study were all purchased from BioLegend (CA, USA) except if otherwise stated: FITC labeled anti-IgM (R6-60.2, BD Biosciences, NJ, USA), anti-CD8 (53-6.7), and anti-IFN-γ (XMG1.2); PE labeled anti-CD8 (53-6.7, BD), anti-CD44 (IM7), anti-CD19 (6D5) and anti-CXCR5 (L138D7); PerCP labeled anti-TNFα (MP6-XT22), anti-CD3 (17A2) and anti-CD4 (RM4-5); PE-Cy7 labeled anti-CD3 (HA2), anti-GL7 (GL7) and anti-PD1 (RMP1-30); APC labeled anti-CD19 (6D5), anti-CD23 (B3B4) and anti-IL-10 (JES5-16E3); BV510 labeled anti-CD4 (RM4-5), PB labeled anti-CD21 (7E9) and anti-IL-2 (JES6-5H4); and BV421 labeled anti-CD62L (MEL-14).

To analyze lymphoid cell populations, different antibody panels were designed. The general lymphoid panel was composed of anti-CD8, -CD3, -CD4, and -CD19. The T cell memory phenotype panel was composed of anti-CD8, -CD3, -CD4, -CD44, and -CD62L. The “T follicular” panel was composed of anti-CD3, -CD4 –PD1, and –CXCR5. The “B cell phenotype” panel was composed of anti-CD19, -IgM, -CD21, -CD23 and -GL7. Surface staining of splenic cells was performed in PBS + 0.5% BSA (20 min, 4°C) followed by 15 min fixation using 1% PFA. For intracellular staining, splenocytes were cultured for 4 h with PMA/Ionomycin (50/500 ng/ml) and Brefeldin A (10 μg/mL). Cells were surface-stained and then intracellularly after fixation and permeabilization with 1% saponin (all from Sigma-Aldrich, MO, USA). Isotype controls were always used for this study. Samples were acquired in a FACSCanto (BD, Franklin Lakes, NJ, USA) and analyzed with FlowJo software v10 (TreeStar, OR, USA).

Supplementary Figure 1 illustrates the gating strategies used in this work. Briefly, an initial gate plotting FSC-A vs. SSC-A was performed to exclude cell debris. Afterward, singlets were selected by plotting FSC-A vs. FSC-H and the remaining cell populations were resolved. T lymphoid cell populations were defined as CD3+/CD4+ and CD3+/CD8+ while B cells were defined as CD19+. Memory populations were defined as Naïve (CD62L+CD44-), T Effector Memory (TEM – CD62L-CD44+), and T Central Memory (TCM – CD62L+CD44+). Expression of CD21, CD23, and GL7 was evaluated within B cells. Cytokine production by T cells was assessed within CD3+/CD4+ and CD3+/CD8+ cells. Co-expression of PD1 and CXCR5 was evaluated within CD3+/CD4+ T cells.

### Immunoglobulin Determination

Antigen-specific immunoglobulins were quantified by sandwich ELISA. Briefly, high protein binding 96-well plates (Greiner Bio-One, Kremsmünster, Austria) were coated overnight at 4°C with Soluble Total *Leishmania infantum* Antigens (Silvestre et al., 2008) prepared in NaHCO3 0.1 M to a final concentration of 40 μg/ml. Plates were then washed with PBS Tween 0.1%, blocked with 1% gelatin from porcine skin (Sigma-Aldrich, MO, USA) in PBS (blocking buffer) for 1 h at 37°C and re-washed. Each serum was then diluted 1:100 in blocking buffer and added to the plates in duplicate. Wells filled with just blocking buffer were used as blanks. Plates were incubated for 2 h at 37°C and re-washed. Afterward, IgG and IgG1 were detected using horseradish peroxidase (HRP) coupled α-mouse antibodies [diluted 1:5,000 (IgG1; Southern Biotech, AL, USA) or 1:8,000 (IgG; Southern Biotech, AL, USA) in blocking buffer; incubated for 1 h, at 37°C]. Plates were washed for the last time, and the substrate (ortho phenyl diamine (OPD) in citrate buffer) was added for 10 min, time after which the reaction was stopped with HCl 3 N. Absorbance values were determined at 492 nm in a Synergy^TM^ 2 Multi-Mode Reader (BioTek instruments, VT, USA).

### Statistical Analysis

Results are expressed per individual animals/samples and/or normalized in relation to the average values of the respective control groups, with a representation of the group mean-value ± standard deviation. Statistical differences were analyzed using GraphPad Prism v6.01 (CA, USA). One Way ANOVA (with Tukey's *post-hoc* analysis) was used for comparisons between the different murine strains (infected or non-infected), as well as for the comparison of normalized values; *t*-test was used for comparison between infected animals and the respective controls.

## Results

### Dynamics of *L. infantum* Infection in the Different Mouse Strains: Looking at the Main Target Organs

**Figure 1:**
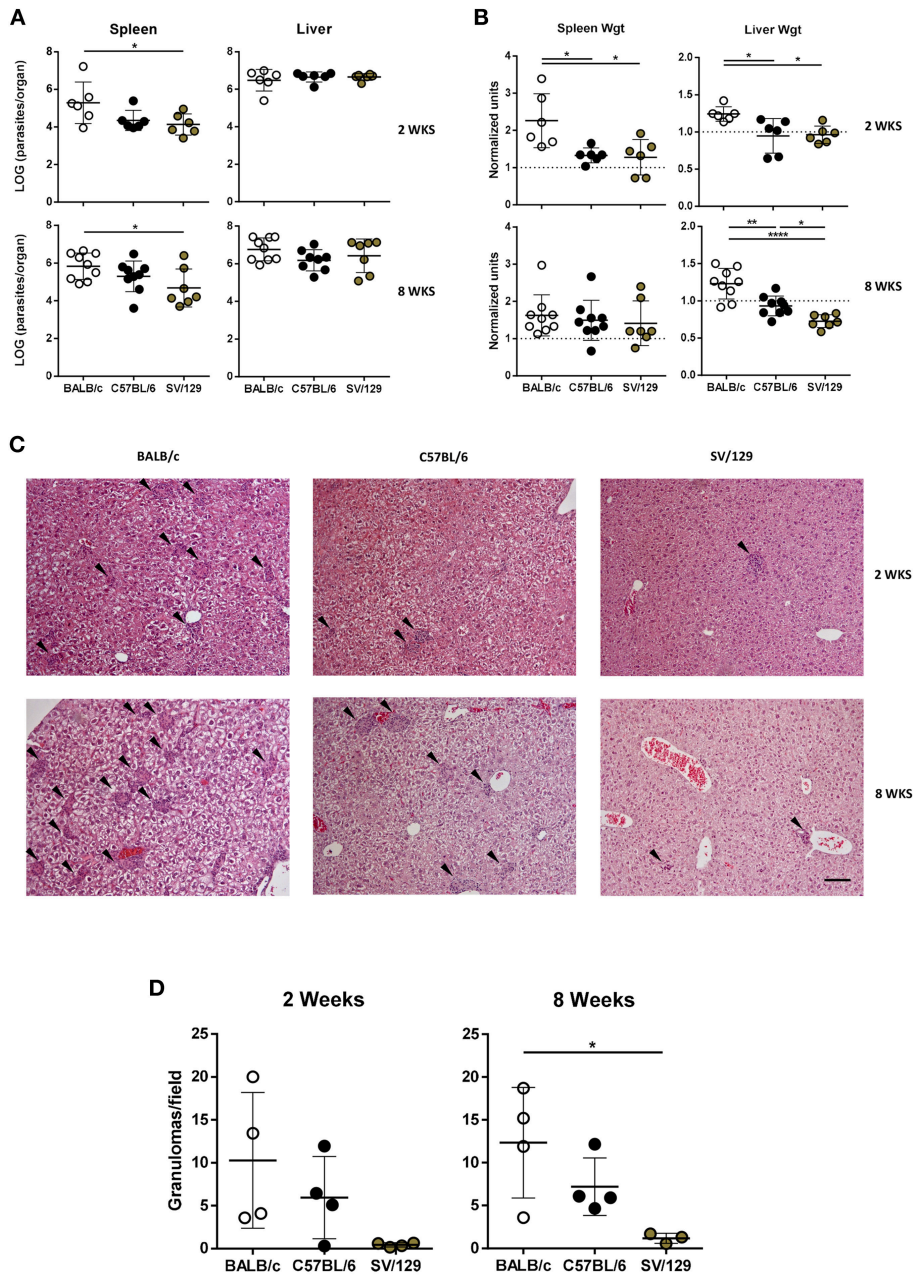
Parasite burdens, organ weights, and hepatic granulomas quantification in the three murine strains 2 and 8 weeks after infection with *L. infantum*. BALB/c (white circles), C57BL/6 (black circles) and SV/129 (brown circles) mice were infected intraperitoneally with 1 × 10^8^ *L. infantum* promastigotes and euthanized 2 or 8 weeks after. Aged matched non-infected controls were euthanized at the same time-points. **(A)** Hepatic and splenic total parasite burdens (determined by limiting dilution). **(B)** Relative splenic and hepatic weight alterations with infection (normalized by the controls average organ weights). **(C)** Hepatic granuloma morphology (representative images of H&E stained liver slides; 100X magnification; arrowheads point to granulomas; scale bar corresponds to 100 μm) and **(D)** quantification. Each dot represents an animal; average and SD of the values within each group are shown **(A,B,D)**. Results are representative of at least two independent experiments. Statistical differences are properly identified (One-Way ANOVA (with Tukey's *post-hoc* analysis): ^*^p ≤ 0.05, ^**^p ≤ 0.01, and ^****^p ≤ 0.0001).

To study the determinants of visceral leishmaniasis resistance vs. susceptibility, we compared the evolution of experimental *L. infantum* infection in three inbred mouse strains. SV/129 is the strain described as resistant while C57BL/6 and BALB/c are defined as susceptible models in the context of *L. donovani* infection (Lipoldova and Demant, 2006). Two weeks after an intraperitoneal challenge with 1 × 10^8^ *L. infantum* promastigotes, BALB/c mice showed significantly higher splenic parasite burdens in comparison with their SV/129 counterparts (Figure 1A, *p* ≤ 0.05). This was accompanied by infection-induced increased hepatic and splenic weights in BALB/c, in a higher magnitude than the one observed for both C57BL/6 and SV/129 (Figure 1B, *p* ≤ 0.05). Eight weeks post-infection, BALB/c mice still presented the highest splenic parasite counts, followed by C57BL/6 and last by SV/129 Figure 1A, *p* ≤ 0.05), although at this time-point relative spleen weights were comparable among all the mouse strains (Figure 1B). Interestingly, at this later time point, relative liver weights were significantly different comparing the mouse strains (at least *p* ≤ 0.05): BALB/c relative liver weight was the highest, while SV/129 was the lowest (Figure 1B) with a liver weight below the determined weight for control animals (Supplementary Figure 2A). However, such differences were not translated into different absolute hepatic parasite burdens, determined comparable among groups at both 2- and 8-weeks post-infection. Curiously, liver granulomatous response to *L. infantum* infection was consistently different between the mouse strains (Figures 1C,D, Supplementary Figure 2B). While BALB/c mice liver-sections contained on average 10 granulomas/field at both 2- and 8-weeks post-infection, in C57BL/6 we observed on average five hepatic granulomas/field (2- and 8 -weeks post-infection) and in SV/129 always less than five granulomas/field (Figure 1D). Additionally, we observed that SV/129 had rather less structured cell infiltrates when compared to the more susceptible mouse strains (Figure 1C).

### Basal Differences in the Murine Strains Splenic Cell Compartments and Their Modification 2 Weeks Post-infection

**Figure 2:**
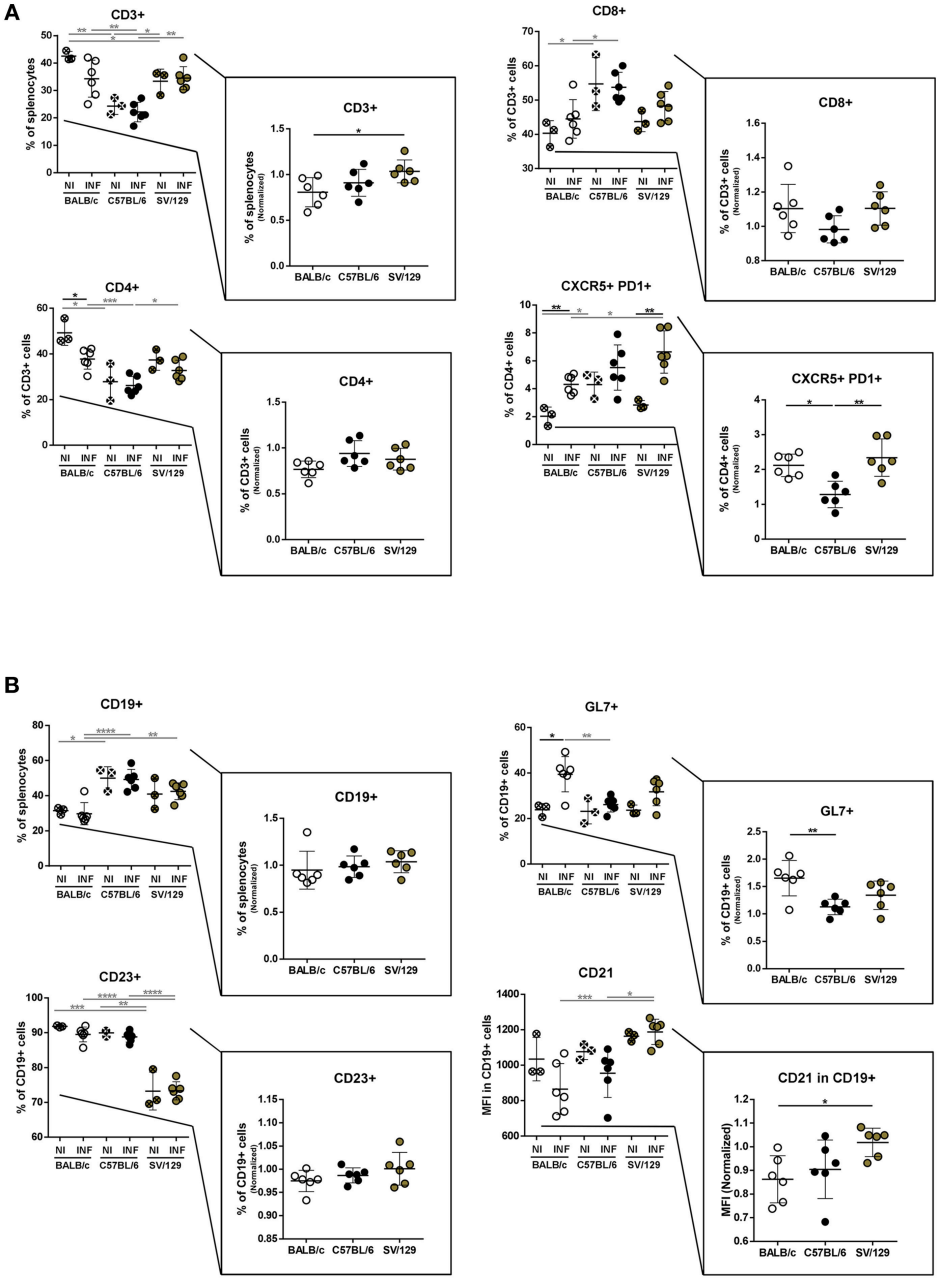
Splenic lymphoid compartment of the different mouse strains and it's alteration 2 weeks post *L. infantum* infection. BALB/c (white circles), C57BL/6 (black circles), and SV/129 (brown circles) mice were infected intraperitoneally with 1 × 10^8^ *L. infantum* promastigotes and euthanized 2 weeks after. Splenic lymphoid populations were resolved by flow cytometry: T cell **(A)** and B cell **(B)** compartments. Results, obtained in at least two independent experiments, are represented both in total percentages (infected and control animals) and normalized (infected in relation to control group average values) as a way to highlight the infection-induced alterations. Each dot represents an animal. Average and SD of the values within each group are shown. Statistical differences are properly identified. One Way ANOVA (with Tukey's *post-hoc* analysis) was used for comparisons between the different murine strains (infected or non-infected; gray lines), as well as for comparison of normalized values (black lines); *t*-tests (black lines) were performed for comparison between infected animals and controls from the same strain: ^*^p ≤ 0.05, ^**^p ≤ 0.01, ^***^p ≤ 0.001, and ^****^p ≤ 0.0001).

It is known that the splenic cell compartments of different murine strains are not similar (Forni, 1988). To understand if such differences would influence the early response to *L. infantum* infection, we resolved by flow cytometry the splenic cell compartments of the three murine strains studied, comparing animals infected for 2 weeks with their non-infected counterparts. While regarding the lymphoid splenic cell populations, we observed differences comparing BALB/c, C57BL/6, and SV/129 both before and 2 weeks after infection (Figures 2A,B), this was not true for myeloid cell populations for which no major differences were observed (data not shown).

T lymphocytes (CD3+) represented more than 35% of spleen cells in BALB/c and SV/129 control mice, while in C57BL/6 this population accounted in average for 25% of this organ basal cell content (Figure 2A). Furthermore, within splenic T lymphocytes the basal levels of CD4+ cells were higher in BALB/c (50%) than in SV/129 (40%) and C57BL/6 (30%), while the basal levels of CD8+ were higher in C57BL/6 (55%) compared to both BALB/c and SV/129 mice (around 40%) (Figure 2A). Two weeks post-infection, these general tendencies were overall maintained, although an infection-induced effect was observed for BALB/c mice: CD3+ T cells decreased with infection (statistically different normalized numbers compared with SV/129, *p* ≤ 0.05), as well as CD4+ T cells (*p* ≤ 0.05 compared with non-infected values). We also resolved the T cell memory pool, and although we observed some differences comparing the strains, no major infection-induced effect was observed for CD4+ T cells, while for CD8+ T cells an infection-induced decrease in naïve and an increase in TEM and TCM populations was observed, particularly comparing C57BL/6 with BALB/c animals (Supplementary Figure 3). Additionally, we analyzed a subset of CD4+ T lymphocytes expressing CXCR5 and PD1, known as follicular helper T cells (Tfh), important for germinal center reaction related to T-B cell cognate interaction, since they may play an important role in the anti-*Leishmania* immune response (Rodrigues et al., 2014). Although the basal levels of this population were higher in C57BL/6 animals, 2 weeks post-infection this CD4 subset significantly increased in BALB/c and SV/129 mice, while it did not change in C57BL/6 mice (Figure 1A, *p* ≤ 0.05 or *p* ≤ 0.01, compared with BALB/c or SV/129, respectively).

Concerning total splenic B cells (CD19+) we observed that their basal levels in BALB/c mice were lower in comparison with C57BL/6 animals (Figure 2B). Furthermore, no major changes were observed in the frequencies of splenic B cells 2 weeks after infection (Figure 2B). Although most of these B cells expressed CD23 [important down-regulator of BCR signaling (Liu et al., 2016)], SV129 displayed a relatively larger population of CD19+CD23lo/- cells than BALB/c or C57BL/6. However, once again no significant changes in the frequency of CD19+CD23+ cells were observed as a consequence of *L. infantum* infection. CD21 is an important co-receptor molecule in BCR cognate stimulation (Roozendaal and Carroll, 2007). Although no significant differences were detected in terms of basal expression, comparing the three murine strains, a downregulation of this receptor 2 weeks after infection was detected for both BALB/c and C57BL/6 (Figure 2B), but not for SV129. Last but not least, we also observed a significant increase in the expression of the activation marker GL7 only in BALB/c mice 2 weeks after infection (Figure 2B; *p* ≤ 0.05).

### Murine Strains Splenic Cell Compartment Composition 8 Weeks Post-infection

**Figure 3:**
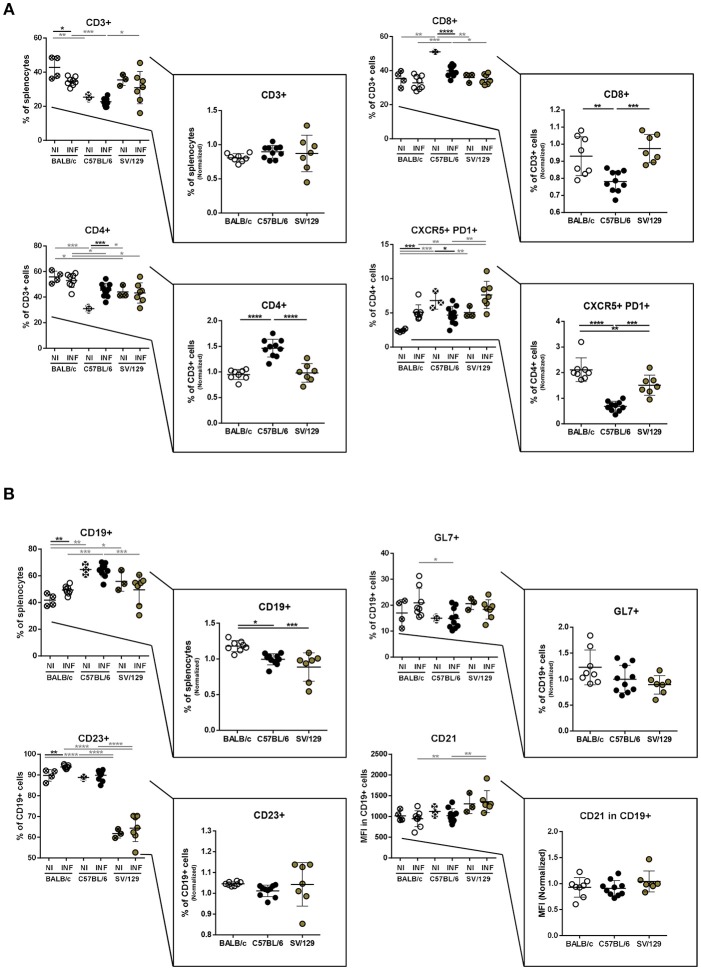
Splenic lymphoid compartment of the different mouse strains and it's alteration 8 weeks post *L. infantum* infection. BALB/c (white circles), C57BL/6 (black circles), and SV/129 (brown circles) mice were infected intraperitoneally with 1 × 10^8^ *L. infantum* promastigotes and euthanized 8 weeks after. Splenic lymphoid populations were resolved by flow cytometry: T cell **(A)** and B cell **(B)** compartments. Results, obtained in at least two independent experiments, are represented both in total percentages (infected and control animals) and normalized (infected in relation to control group average values) as a way to highlight the infection-induced alterations. Each dot represents an animal. Average and SD of the values within each group are shown. Statistical differences are properly identified. One Way ANOVA (with Tukey's *post-hoc* analysis) was used for comparisons between the different murine strains (infected or non-infected; gray lines), as well as for comparison of normalized values (black lines); *t*-tests (black lines) were performed for comparison between infected animals and controls from the same strain: ^*^p ≤ 0.05, ^**^p ≤ 0.01, ^***^p ≤ 0.001, and ^****^p ≤ 0.0001).

To figure out if the murine strain's splenic cell compartments would differentially change with the course of *L. infantum* infection, we performed flow cytometry analysis in the spleens from age-matched BALB/c, C57BL/6 and SV/129 mice, at 8 weeks post-infection, always in comparison with the respective non-infected and age-matched controls. Once again, in the myeloid cell compartment no major infection-induced differences were seen at this later time-point (data not shown) while in the lymphoid cell compartment some alterations with the course of infection were observed (Figure 3). The CD3+ cells normalized levels at this time point were comparable among the groups, while the CD4+ and CD8+ normalized values were significantly different in C57BL/6 mice compared to both BALB/c and SV/129 strains: the infection induced an increase of CD4+ and a decrease of CD8+ T cells (Figure 3A). Furthermore, with respect to the T cell memory pool, apart from the previously observed strain-specific differences in basal levels, no major infection-induced alterations were observed (Supplementary Figure 4). Additionally, and as observed 2 weeks after infection, differences in CD4+CXCR5+PD1+ T cells were detected, comparing the different mouse strains, regarding both basal levels and the ones determined in 8-week-infected animals. While C57BL/6 mice showed a decrease of splenic Tfh cells (*p* ≤ 0.05; compared with control counterparts), SV/129, and BALB/c mice showed an increase in this splenic T cell subset, more pronounced for the latest (Figure 3A; *p* ≤ 0.001, compared with control counterparts). Finally, at this later time-point, an infection-induced increase in the splenic B cells was observed in BALB/c mice (*p* ≤ 0.01) but not in C57BL/6 nor SV/129 animals, although phenotypically no alterations were observed with infection comparing the different mouse strains (Figure 3B).

### Dynamics of Splenic T Cell's Cytokine Response With the Course of Infection in the Different Mouse Strains

It is well-known that the inflammatory environment of target organs conditions parasite persistence or elimination (Rodrigues et al., 2016). These responses are particularly relevant in the spleen, organ used many times to distinguish between susceptible and resistant animal models, related to observations of infection progression or parasite elimination, respectively (Stanley and Engwerda, 2007). To extrapolate the infection-induced environment in the spleen of the different animal models used in this study, and to disclose potential differences between them, we evaluated by flow cytometry the cytokine production potential of splenic T cells at 2- and 8-weeks post-infection, always compared to non-infected matched controls.

**Figure 4:**
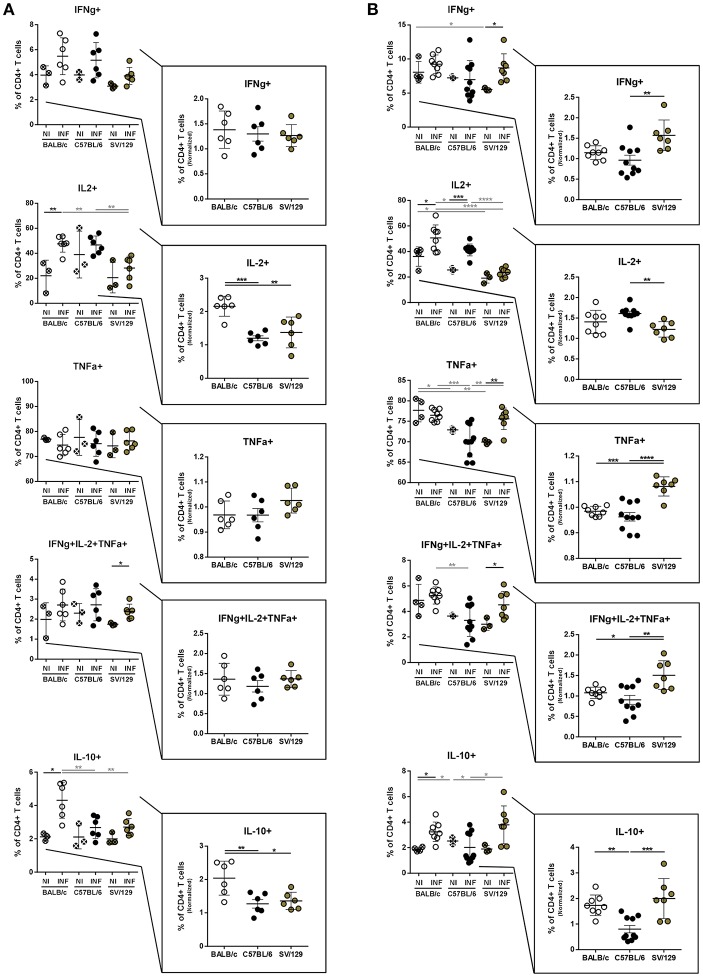
Basal intracellular cytokine production by CD4+ T cells of the different mouse strains and it's alteration 2- and 8-weeks post *L. infantum* infection. BALB/c (white circles), C57BL/6 (black circles), and SV/129 (brown circles) mice were infected intraperitoneally with 1 × 10^8^ *L. infantum* promastigotes and euthanized 2 or 8 weeks after. Splenocytes were cultured with PMA + Ionomycin + Brefeldin A for 4 h. CD4+ T cell frequencies producing IFN-γ, IL-2, TNF-α, and IL-10 were resolved by flow cytometry and are denoted for 2 **(A)** and 8 **(B)** weeks-infected animals and their respective controls. Results, obtained in at least two independent experiments, are represented both in total percentages (infected and control animals) and normalized (infected in relation to control group average values) as a way to highlight the infection-induced alterations. Each dot represents an animal. Average and SD of the values within each group are shown. Statistical differences are properly identified. One Way ANOVA (with Tukey's *post-hoc* analysis) was used for comparisons between the different murine strains (infected or non-infected; gray lines), as well as for comparison of normalized values (black lines); *t*-tests (black lines) were performed for comparison between infected animals and controls from the same strain: ^*^p ≤ 0.05, ^**^p ≤ 0.01, ^***^p ≤ 0.001, and ^****^p ≤ 0.0001).

Overall, no major differences were observed in the basal cytokine production by CD4+ T cells. Additionally, early after infection, all murine strains showed a similar capacity to produce IFN-γ. On the other hand, CD4+ T cells from infected BALB/c mice responded by producing significantly more IL-2 and IL-10 compared to both non-infected counterparts and infected C57BL/6 and SV/129 mice (Figure 4A; at least *p* ≤ 0.05). No significant differences with respect to TNF-α producing CD4+ T cells were observed at this time-point, when comparing the different mouse strains, and consequently, no significant differences were observed when we looked at the frequencies of splenic polyfunctional CD4+ T cells (with the potential to produce simultaneously IFN-γ, IL-2, and TNF-α) (Figure 4A). However, 8 weeks after infection, SV/129 mice displayed a significant increase in the percentage of splenic CD4+ T cells with the capacity to secrete IFN-γ or TNF-α (Figure 4B; at least *p* ≤ 0.05). This translated into a significant increase in the levels of splenic polyfunctional CD4+ T cells, comparing SV/129 animals with both BALB/c and C57BL/6 mice (Figure 4B; *p* ≤ 0.05 and *p* ≤ 0.01, respectively). Furthermore, at 8 weeks post-infection, the percentage of splenic CD4+ T cells showing the potential to secrete IL-10 was significantly and tendentiously increased in BALB/c and SV129 mice, respectively. Curiously, at this later time-point, the only infection-induced phenotype observed for C57BL/6 animals was the significant increase in the IL-2 production by splenic CD4+ T cells (Figure 4B).

**Figure 5:**
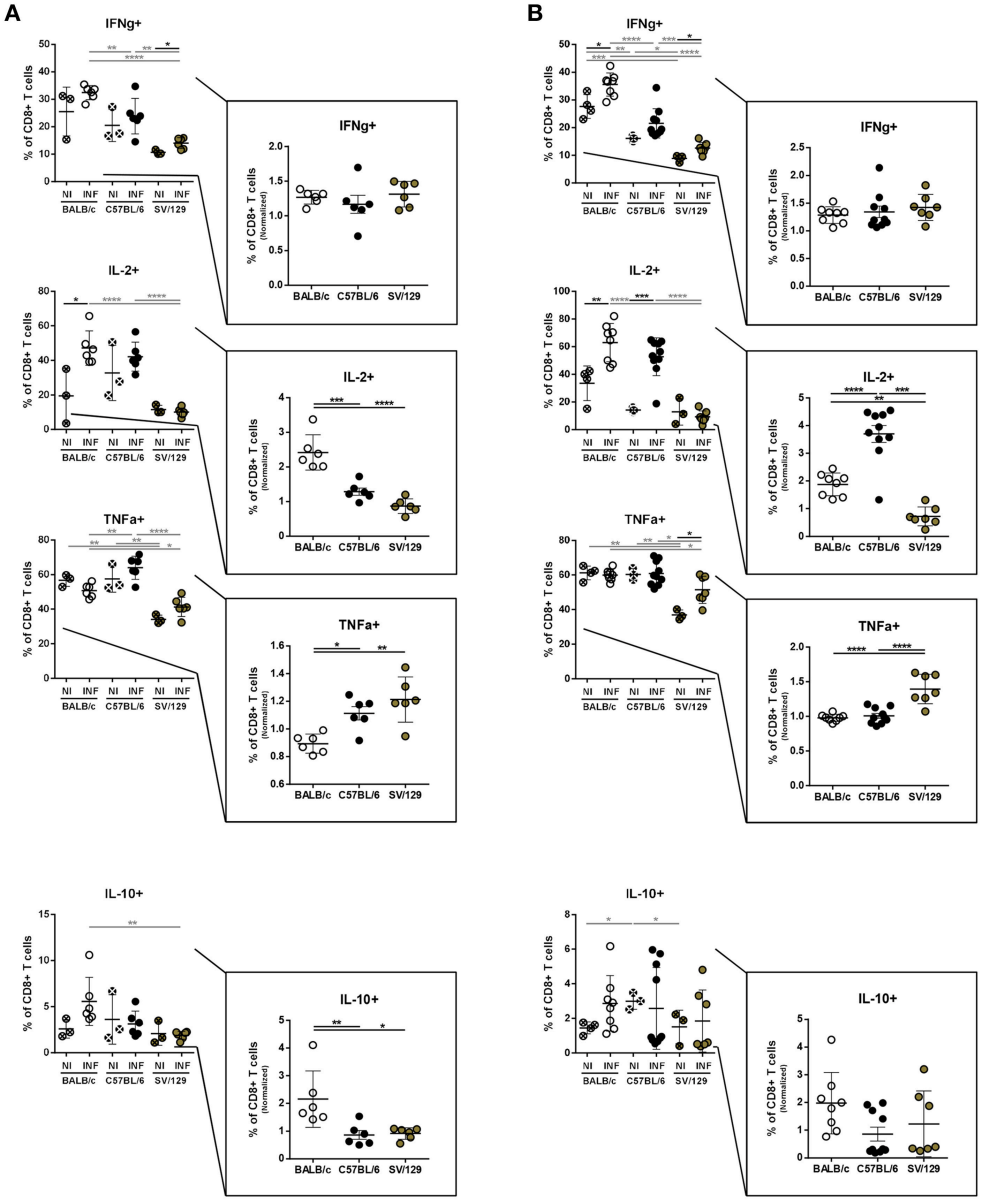
Basal intracellular cytokine production by CD8+ T cells of the different mouse strains and it's alteration 2- and 8-weeks post *L. infantum* infection. BALB/c (white circles), C57BL/6 (black circles) and SV/129 (brown circles) mice were infected intraperitoneally with 1 × 10^8^ *L. infantum* promastigotes and euthanized 2 or 8 weeks after. Splenocytes were cultured with PMA + Ionomycin + Brefeldin A for 4 h. CD8+ T cell frequencies producing IFN-γ, IL-2, TNF-α, and IL-10 were resolved by flow cytometry and are denoted for two **(A)** and 8 **(B)** weeks-infected animals and their respective controls. Results, obtained in at least two independent experiments, are represented both in total percentages (infected and control animals) and normalized (infected in relation to control group average values) as a way to highlight the infection-induced alterations. Each dot represents an animal. Average and SD of the values within each group are shown. Statistical differences are properly identified. One Way ANOVA (with Tukey's *post-hoc* analysis) was used for comparisons between the different murine strains (infected or non-infected; gray lines), as well as for comparison of normalized values (black lines); *t*-tests (black lines) were performed for comparison between infected animals and controls from the same strain: ^*^p ≤ 0.05, ^**^p ≤ 0.01, ^***^p ≤ 0.001, and ^****^p ≤ 0.0001).

Regarding the basal splenic CD8+ T cell cytokine-secreting potential, some differences were observed, particularly comparing SV/129 with the other models that show a higher response (Figure 5A). However, when we evaluated infection-induced effects 2 weeks post-challenge, splenic CD8+ T cells from C57BL/6 and SV/129 mice showed a greater potential of TNF-α secretion, when compared to BALB/c animals (Figure 5A; at least *p* ≤ 0.05 comparing normalized values). Once again at this early time-point after infection, no major differences were observed in terms of infection-induced IFN-γ secretion (now by CD8+ T cells), while a significant increase in the splenic CD8+ T cells with the ability to produce either IL-10 or IL-2 was observed comparing BALB/c mice with both C57BL/6 and SV/129 animals (Figure 5A; at least *p* ≤ 0.05 comparing normalized values). At 8 weeks post-infection we once again detected some differences in CD8+ T cell's cytokine-secretion potential when we compared the three mouse strains. While the infection-induced increase of CD8+ T cells producing IFN-γ was not strain related, TNF-α splenic CD8+ T cell secretion potential of SV/129 mice was significantly higher than the one of CD8+ T cells from both BALB/c and C57BL/6 animals (Figure 5B; *p* ≤ 0.0001). Furthermore, as for CD4+ T cells at this time-point, splenic CD8+ T cells from C57BL/6 mice showed a significantly higher capacity of producing IL-2 than both CD8+ T cells from BALB/c and SV/129 (Figure 5B; at least *p* ≤ 0.001). Finally, at this later time-point post-infection, CD8+ T cells from infected BALB/c mice maintained their capacity to secrete IL-10 (Figure 5B).

### Anti-*Leishmania* Specific Immunoglobulin Response to Infection in the Three Murine Strains

**Figure 6:**
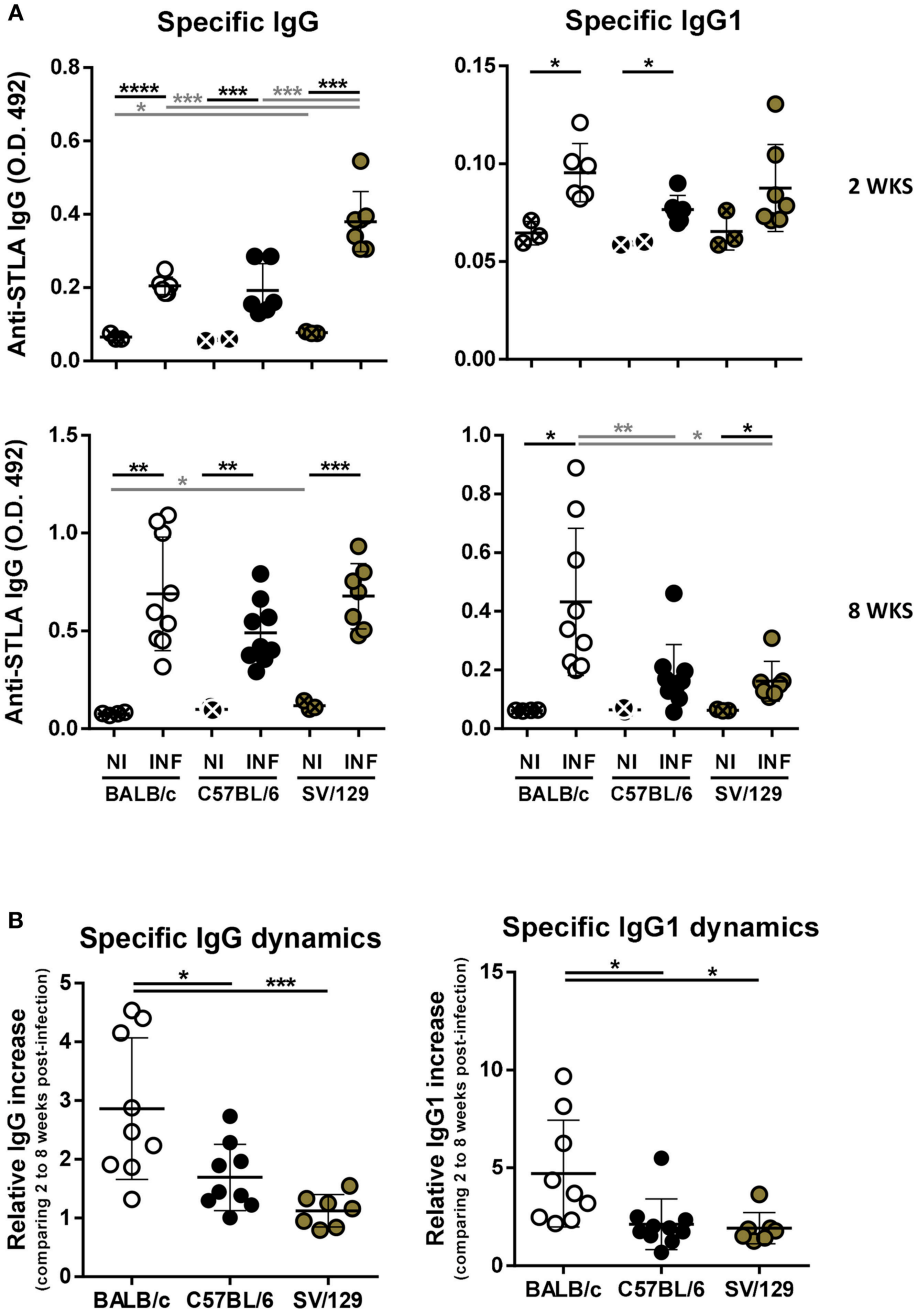
Specific humoral response dynamics in the different *L. infantum* infection murine models. BALB/c (white circles), C57BL/6 (black circles), and SV/129 (brown circles) mice were infected intraperitoneally with 1 × 10^8^ *L. infantum* promastigotes and euthanized 2 or 8 weeks after. **(A)** Specific seric IgG and IgG1 levels against parasite's soluble extract were determined by ELISA for the different infection models and the respective controls in the two time-points evaluated. **(B)** Specific immunoglobulin dynamics was calculated dividing the values obtained for 8 weeks-infected animals (normalized against the respective controls) by the average of the same values obtained for 2 weeks-infected animals. Results are representative of at least two independent experiments. Each dot represents an animal. Average and SD of the values within each group are shown. Statistical differences are properly identified. One Way ANOVA (with Tukey's *post-hoc* analysis) was used for comparisons between the different murine strains (infected or non-infected; gray lines), as well as for comparison of normalized values (black lines); *t*-tests (black lines) were performed for comparison between infected animals and controls from the same strain: ^*^p ≤ 0.05, ^**^p ≤ 0.01, ^***^p ≤ 0.001, and ^****^p ≤ 0.0001).

The role of antibodies in leishmaniasis susceptibility vs. resistance is still not clear (Rodrigues et al., 2016). However, antibody responses, particularly having in consideration IgG isotypes, as the switch for IgG1 isotype is dependent on IL-4 and can be a marker of a Th2 response, may help us to better understand the immune response being mounted against *Leishmania* parasites (Tripathi et al., 2007). Because of this, we evaluated, using ELISA, the specific antibody responses generated upon *Leishmania* infection in the three mouse strains, at 2- and 8-weeks post-infection. Specific IgG response could be detected 2 weeks after *L. infantum* infection in mice from the three strains (Figure 6A). Curiously, SV/129 mice presented significantly higher levels of serum IgG that binds to *L. infantum* antigens, in comparison with both BALB/c and C57BL/6 strains (Figure 6A; *p* ≤ 0.001). Importantly, this difference was not mediated by a significant increase of anti-*Leihmania* specific IgG1 antibodies (Figure 6A). Later, 8 weeks after infection, we did not observe any further difference in the levels of serum *L. infantum* specific IgG antibodies, comparing all mouse strains. However, at this later time-point, BALB/c mice presented significantly higher levels of serum *L. infantum* specific IgG1 antibodies, when compared to both C57BL/6 and SV129 infected mice (Figure 6A; at least *p* ≤ 0.05). These results were evidenced by the calculation of specific immunoglobulin dynamics: BALB/c mice presented on average 5-fold more anti-*Leihmania* specific IgG1 antibodies at 8 vs. 2 weeks post-infection, while C57BL/6 and SV129 mice presented no more than a 2-fold increase.

## Discussion

One of the layers of complexity associated with leishmaniasis relates to the distinct known disease manifestations, a consequence of heterogeneous pathogen-host interactions that will condition the infectious process outcome (Loeuillet et al., 2016; Cecilio et al., 2018). While for some disease forms, such as cutaneous leishmaniasis, we are close to understanding the determinants of resistance vs. susceptibility, for visceral leishmaniasis we are still unable to clearly establish this dichotomy. Here, trying to address this issue, we took advantage of different inbred mouse models, with known different susceptibility patterns to *Leishmania* spp. infection, and dynamically explored their splenic cell compartment's composition, in health, and after infection with *L. infantum*. Furthermore, we also looked at local and systemic infection-induced immune responses, comparing the different murine strains.

As expected, pathological differences were observed when we compared the different infected mouse strains. Two weeks post-infection BALB/c mice demonstrated hepatosplenomegaly, while both C57BL/6 and SV/129 mice kept their splenic and hepatic weights comparable to the respective controls (Figure 1B). Eight weeks post-infection, BALB/c mice continued to be the only model demonstrating hepatomegaly, while all of the strains showed similar signs of splenomegaly (Figure 1B). Interestingly, determined hepatic weights were in accordance with the granulomatous response quantified (Figures 1C,D), which may indicate a correlation between cell infiltrates and hepatic weight.

The above-mentioned results partially translated to the determined parasite burdens, particularly considering the splenic ones. As early as 2 weeks post-infection, we detected a significant difference when comparing the susceptible BALB/c model with the resistant SV/129 (Figure 1A). These results are in line with the ones reported for the other viscerotropic *Leishmania* species, *L. donovani* (Bodhale et al., 2018). Interestingly, similar to what (Bodhale et al., 2018) reported, we also observed an increase of splenic parasite burdens with time in BALB/c and C57BL/6 mice, while for SV/129 mice we observed a plateau. Additionally, also similar with *L. donovani* infection, the splenic parasite burdens of C57BL/6 animals were always intermediate, compared with the other two animal models (Bodhale et al., 2018). Overall this may suggest that the pathogenic process of these two different parasite strains is similar, as will probably be the determinants of susceptibility vs. resistance. This gives relevance to the convergent study of the two viscerotropic *Leishmania* spp. *in vivo*, as a way to tackle the many questions that still remain unanswered, that go beyond the dichotomy susceptibility vs. resistance (e.g., determinants of visceralization).

Regarding liver parasite burdens, no significant differences were detected among the mouse strains at both 2- and 8-weeks post-infection (Figure 1A). Most certainly, at a later time point post-infection, we would observe a reduction in hepatic parasite burdens, considering the organ-specific immunity associated with murine *Leishmania* infection models (Stanley and Engwerda, 2007), together with a milder granulomatous response. Although such a hepatic resolution of infection is observable at early time-points in other studies, we have to keep in mind that *Leishmania* spp. *in vivo* infection outcomes depend on a number of factors, including the parasite strain, the parasite dose, and the route of challenge (Loeuillet et al., 2016).

To understand if the infectious process differentially influences the immune response of different hosts and to try to infer how the host responds to the parasite, we resolved the splenic cell compartments of the three murine strains at two time-points after infection, always comparing with their basal (non-infected) statuses. And more than to identify the main cell types analyzed in host-*Leishmania* interaction studies, we looked at some functional markers of T and B cells. Interestingly, both BALB/c and SV/129 showed higher levels of infection-induced splenic Tfh cells than C57BL/6 mice (Figures 2A, 3A). One possible explanation for such differences may be related to the generation, or absence thereof, of an environment favorable for Tfh differentiation, dependent, among other factors on cytokine secretion (Ma et al., 2012). Curiously, upon *Leishmania infantum* infection, BALB/c but not C57BL/6 mice, showed increased levels of IL-27 (Perez-Cabezas et al., 2016), one of the cytokines associated with Tfh differentiation (Ma et al., 2012). We may only speculate that the infection-induced expansion of the SV/129 Tfh cell compartment may be due to the same infection-induced IL-27 secretion observed in BALB/c mice. Regarding B cell functional markers, the infection-induced differences observed 2 weeks post-infection (comparing the different mouse strains) were absent at the 8 weeks post-infection time-point (Figures 2B, 3B). Relevantly, 2 weeks post-infection, while splenic B cells from SV/129 mice retained the basal levels of CD21 expression, the ones from both BALB/c and C57BL/6 mice showed a downregulation of this surface marker, which may indicate a “more immature” B cell compartment (Thorarinsdottir et al., 2015). Such results are fittingly in line with the specific antibody titers determined against total parasite extract (Figure 6A), significantly higher in SV/129 mice early after infection, in comparison with the other two murine strains. This relation between CD21 expression levels and specific antibody titers is well-characterized in other infection models (Haas et al., 2002; Schauer et al., 2003). These results suggest that SV/129 are faster than BALB/c and C57BL/6 mice in the assembly of an efficient B-cell response that cannot be separated from a competent T-cell response (Crotty, 2015).

To disclose if possible, strain-related characteristics of the infection-induced splenic environment might justify the resistant vs. susceptible phenotypes, we further explored the dynamics of splenic T cell's cytokine response with the course of infection in the different mouse strains. It is well-known that IL-10 regulates the kinetics of visceral *Leishmania* infection (Nylén and Sacks, 2007). Relevantly, here we show that only CD4+ and CD8+ cells from BALB/c mice showed an infection-induced increase capacity of secreting IL-10, which is retained at 8 weeks post-infection (Figures 4, 5). This is probably the main indirect justification of the differences in splenic parasite burdens determined, comparing BALB/c and SV/129 animals. On the other hand, and although also considered a susceptible model of VL, C57BL/6 mice never showed this IL-10 upregulation, while the resistant SV/129 mice showed an infection-induced IL-10 upregulation (in CD4+ T cells only) 8 weeks post-infection. Interestingly, when we looked at polyfunctional CD4+ T cells (Seder et al., 2008) we observed a significant increase in their frequency at 8 weeks post-infection in SV/129 mice compared to the susceptible murine models (Figures 4, 5). Looking at the bigger picture, we may speculate that the control of infection observed in SV/129 mice is associated with this probable increase of T cell polyfunctionality, which is not well-explored in visceral leishmaniasis [only in a few vaccine studies, e.g., (Coler et al., 2015)], but is well-understood in other infectious diseases, such as the one caused by HIV-2 (Duvall et al., 2008). The infection-induced increased potential of IL-10 secretion detected for CD4+ T cells of SV/129 mice may also be an indirect indication of an effective anti-parasitic response, bearing in mind that accompanied with inflammation, regulatory mechanisms are expected to occur in order to limit tissue damage, and ultimately restore homeostasis (Iyer and Cheng, 2012).

Finally, we explored the specific anti-*Leishmania* humoral responses, also dynamically. Curiously, in BALB/c we observed a prevalent IgG1 response that increased with the course of infection (Figure 6B). It is certain that the Th1/Th2 paradigm explains resistance vs. susceptibility in cutaneous disease, but is not that straightforward in the visceral form of leishmaniasis (Wilson et al., 2005; Tripathi et al., 2007). This said, our results support the development of a prevalent Th2-like response in BALB/c mice, but not in C57BL/6 [as expected (Watanabe et al., 2004)] or SV/129 mice, a potential justification of the results observed regarding splenic parasite burdens.

Overall, our results suggest that C57BL/6 mice demonstrates an intermediate “infection-phenotype” compared to the susceptible and resistant BALB/c and SV/129 mouse strains respectively, in the context of *L. infantum* infection. It is difficult to draw a parallel between visceral leishmaniasis mouse models and human disease since the clinicopathological features of the last, are not recapitulated by the first (Loría-Cervera and Andrade-Narváez, 2014). Still, it is proposed by some authors that murine models of visceral disease may be translated to sub-clinical infection (Loría-Cervera and Andrade-Narváez, 2014) hypothesis, which is hard to confirm since most human individuals studied are either active patients or treated individuals. Allowing ourselves some speculation in convergence with this hypothesis, these distinct mouse models may be representative of the spectrum of asymptomatic individuals: those with a higher probability to develop disease (BALB/c), those that remain asymptomatic (C57BL/6) and those that are able to resolve the infection (SV/129).

The genetic determinants of susceptibility vs. resistance in mouse models of visceral leishmaniasis, such as NRAMP functionality are well-known (Lipoldova and Demant, 2006; Loeuillet et al., 2016). However, we believe that regardless of the genetic background [which will obviously condition susceptibility/resistance to disease in humans (Blackwell, 1996), although not entirely], we need to understand the anti-parasitic immune response mounted by susceptible and resistant models of infection, which may or may not be influenced by the identified genetic susceptibility/resistance determinants, since infection establishment depends on a(n) (in)balance of parasite multiplication and elimination.

## Data Availability

All relevant data are within the paper and its supplementary materials.

## Ethics Statement

This study was carried out in accordance with the principles of the Basel Declaration and recommendations of the i3S Animal Ethics Committee and the Portuguese National Authorities for Animal Health guidelines, according to FELASA and AAALAC guidelines, and European legislation (63/2010). The protocol was approved by the i3S Animal Ethics Committee. BP-C and AC have accreditation for animal research given by the Portuguese Veterinary Direction (Ministerial Directive 1005/92).

## Author Contributions

BP-C, RV, and AC conceived and designed the experiments. BP-C, TG, and RV performed the experiments. BP-C, PC, TG, FG, RV, and AC analyzed the data. FG and AC contributed with the reagents, materials, and analysis tools. BP-C, PC, RV, and AC wrote the paper. BP-C, PC, TG, FG, RV, and AC critically revised and approved the paper.

### Conflict of Interest Statement

The authors declare that the research was conducted in the absence of any commercial or financial relationships that could be construed as a potential conflict of interest.
