# Supplementary material for: Understanding Resistance vs. Susceptibility in Visceral Leishmaniasis Using Mouse Models of Leishmania infantum Infection
Source: Front Cell Infect Microbiol. 2019 Mar 1;9:30. doi: 10.3389/fcimb.2019.00030 (PMC6407322; doi:10.3389/fcimb.2019.00030)
Supplement: Supplementary file 1 [file Data_Sheet_1.pdf]

Supplementary Material

Understanding resistance *versus* susceptibility in Visceral Leishmaniasis using mouse models of *Leishmania infantum* infection

Begoña Pérez-Cabezas[1,2], Pedro Cecílio[1,2,3], Tiago Bordeira Gaspar[1,4,5,6], Fátima Gärtner[1,7,8] , Rita Vasconcellos[9], and Anabela Cordeiro-da-Silva[1,2,3] *

[1] i3S - Instituto de Investigação e Inovação em Saúde, Universidade do Porto, Porto, Portugal;

[2]Parasite Disease Group, IBMC - Instituto de Biologia Molecular e Celular, Universidade do Porto, Porto, Portugal;

[3]Departamento de Ciências Biológicas, Faculdade de Farmácia da Universidade do Porto (FFUP), Porto, Portugal;

[4]Cancer Signalling & Metabolism Group, IPATIMUP - Institute of Molecular Pathology and Immunology of University of Porto, Porto, Portugal;

[5]Faculdade de Medicina da Universidade do Porto (FMUP), Porto, Portugal;

[6]Instituto de Ciências Biomédicas Abel Salazar (ICBAS), Universidade do Porto, Porto, Portugal;

[7]Department of Molecular Pathology and Immunology, ICBAS, Universidade do Porto, Porto, Portugal;[8]Glycobiology in Cancer Group, IPATIMUP, Universidade do Porto, Porto, Portugal;

[9]Immunobiology Department, Biology Institute, Universidade Federal Fluminense, Niterói-RJ, Brazil.

*** Correspondence:** Prof. Anabela Cordeiro-da-Silva: cordeiro@ibmc.up.pt

Keywords: *Leishmania*, Visceral Leishmaniasis, Mouse models, Susceptibility *versus* Resistance, Immune Regulation

1 Supplementary Figures

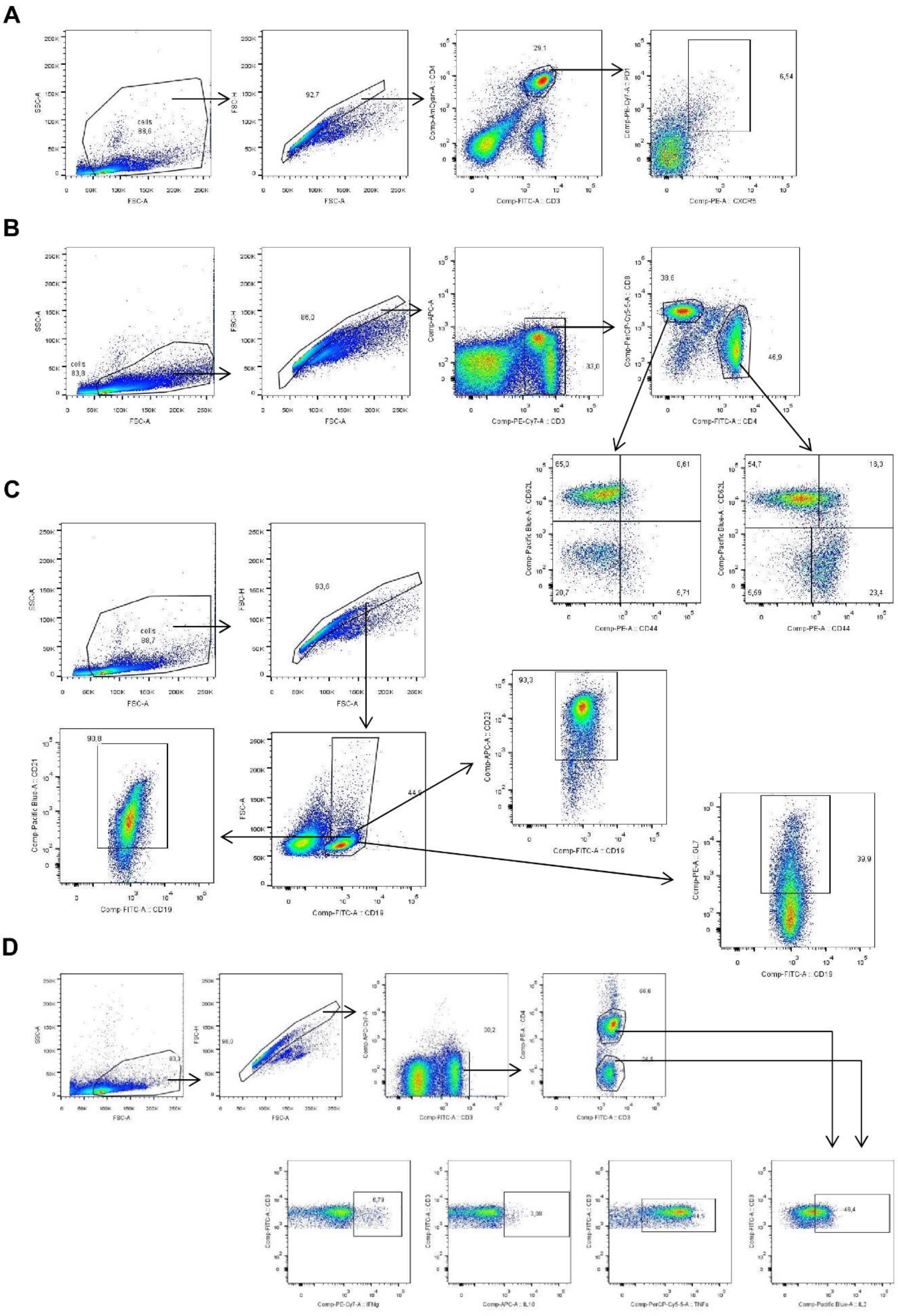

Supplementary figure 1. Gating strategy for flow cytometry analysis. (A) Splenic T follicular helper cells (CD4+CXCR5+PD1+). (B) Splenic T cells and memory phenotype [Naïve (CD62L+CD44-), T Effector Memory (CD62L-CD44+) and T Central Memory (CD62L+CD44+)]. (C) Splenic B cells with analysis of expression of CD21, CD23 and GL7. (D) Evaluation of cytokine production by CD4+ and CD8+ T cells.

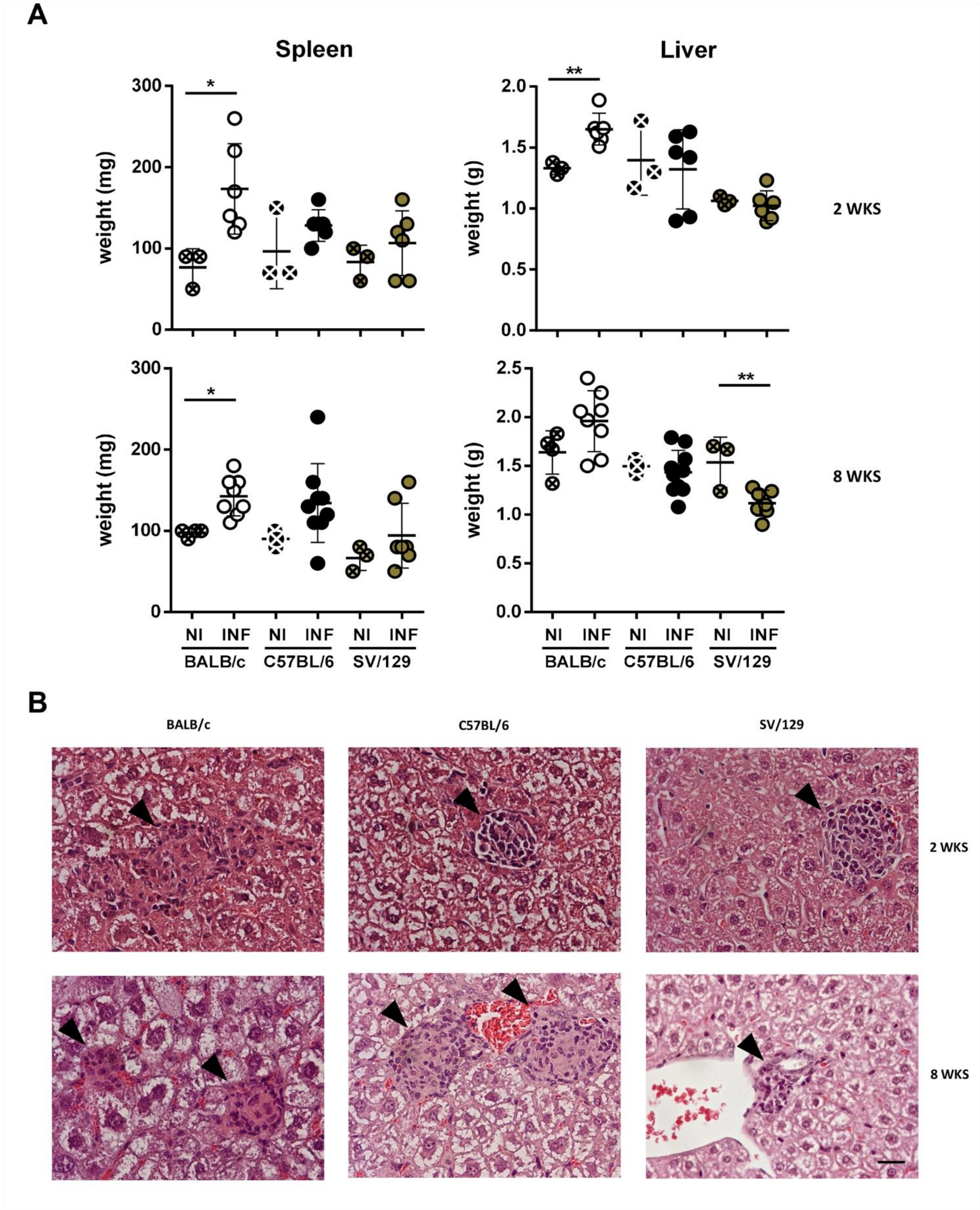

Supplementary figure 2. ***L. infantum*** **infection features of the three murine models 2 and 8 weeks post-challenge: organ weights and granuloma morphology.** BALB/c (white circles), C57BL/6 (black circles) and SV/129 (brown circles) mice were infected intraperitoneally with $1X10^{8}$ *L. infantum* promastigotes and euthanized 2 or 8 weeks after. Aged matched non-infected controls were euthanized at the same time-points. (A) Splenic and hepatic weights. (B) Detailed hepatic granuloma morphology (representative images of H&E stained liver slides; 400X magnification; arrowheads point to granulomas; scale bar corresponds to 25 µm). Results are representative of at least two independent experiments. Each dot represents an animal; average and SD of the values within each group are shown. Statistical differences are properly identified (t-test for comparison between infected animals and controls: * p≤0.05 and ** p≤0.01).

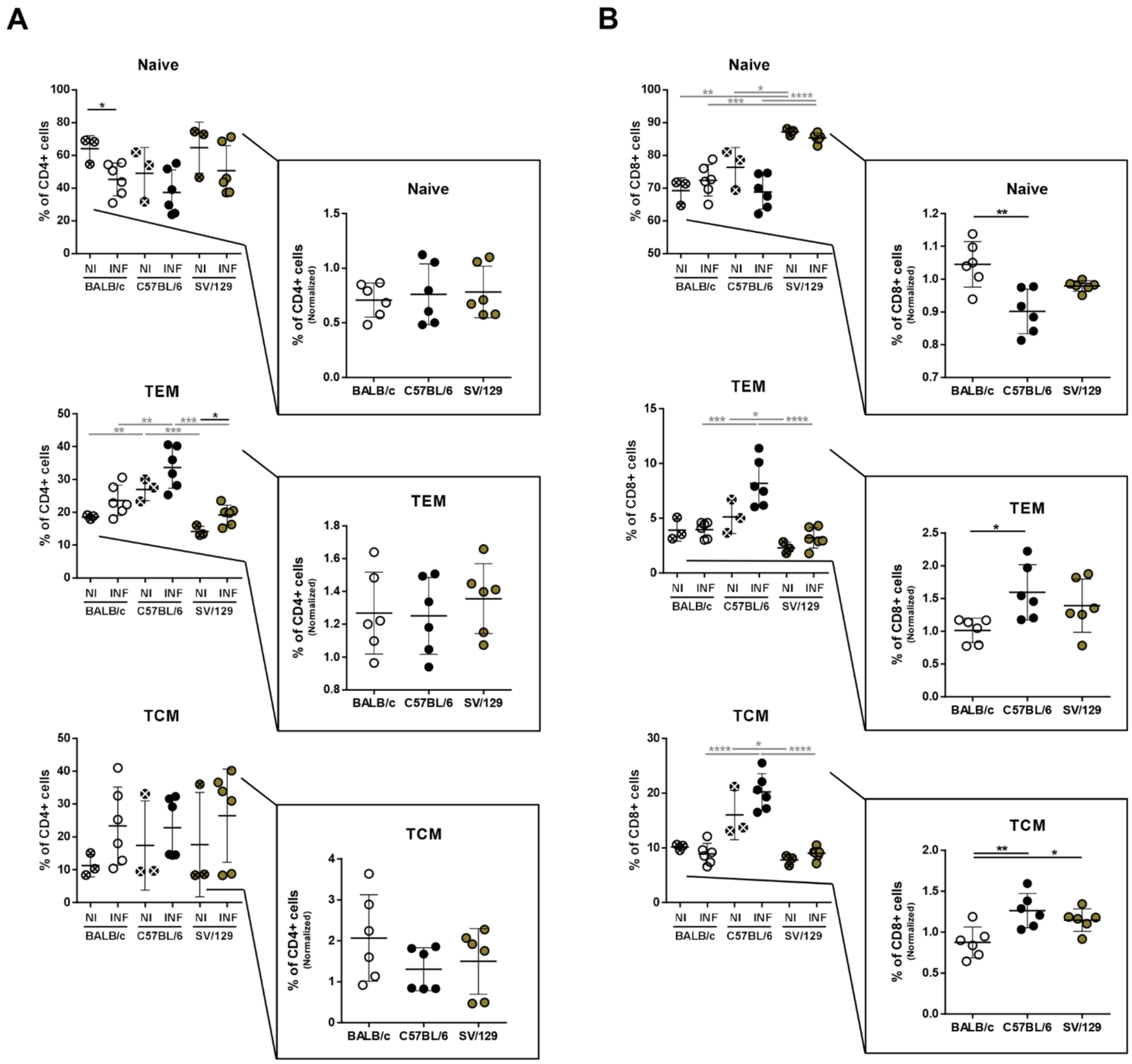

Supplementary figure 3. Splenic T cell memory compartment of the different mice strains and it´s alteration 2 weeks post *L. infantum* infection. BALB/c (white circles), C57BL/6 (black circles) and SV/129 (brown circles) mice were infected intraperitoneally with $1X10^8$ *L. infantum* promastigotes and euthanized 2 weeks after. Splenic CD4+ (A) and CD8+ (B) T cell memory compartment was resolved by flow cytometry based on CD44 and CD62L expression. Results, obtained in at least two independent experiments, are represented both in total percentages (infected and control animals) and normalized (infected in relation to control group average values) as a way to highlight the infection-induced alterations. Each dot represents an animal. Average and SD of the values within each group are shown. Statistical differences are properly identified. One Way ANOVA (with Tukey´s *post hoc* analysis) was used for comparisons between the different murine strains (infected or non-infected; grey lines), as well as for comparison of normalized values (black lines); t-tests (black lines) were performed for comparison between infected animals and controls from the same strain: * p≤0.05, ** p≤0.01, *** p≤0.001 and **** p≤0.0001).

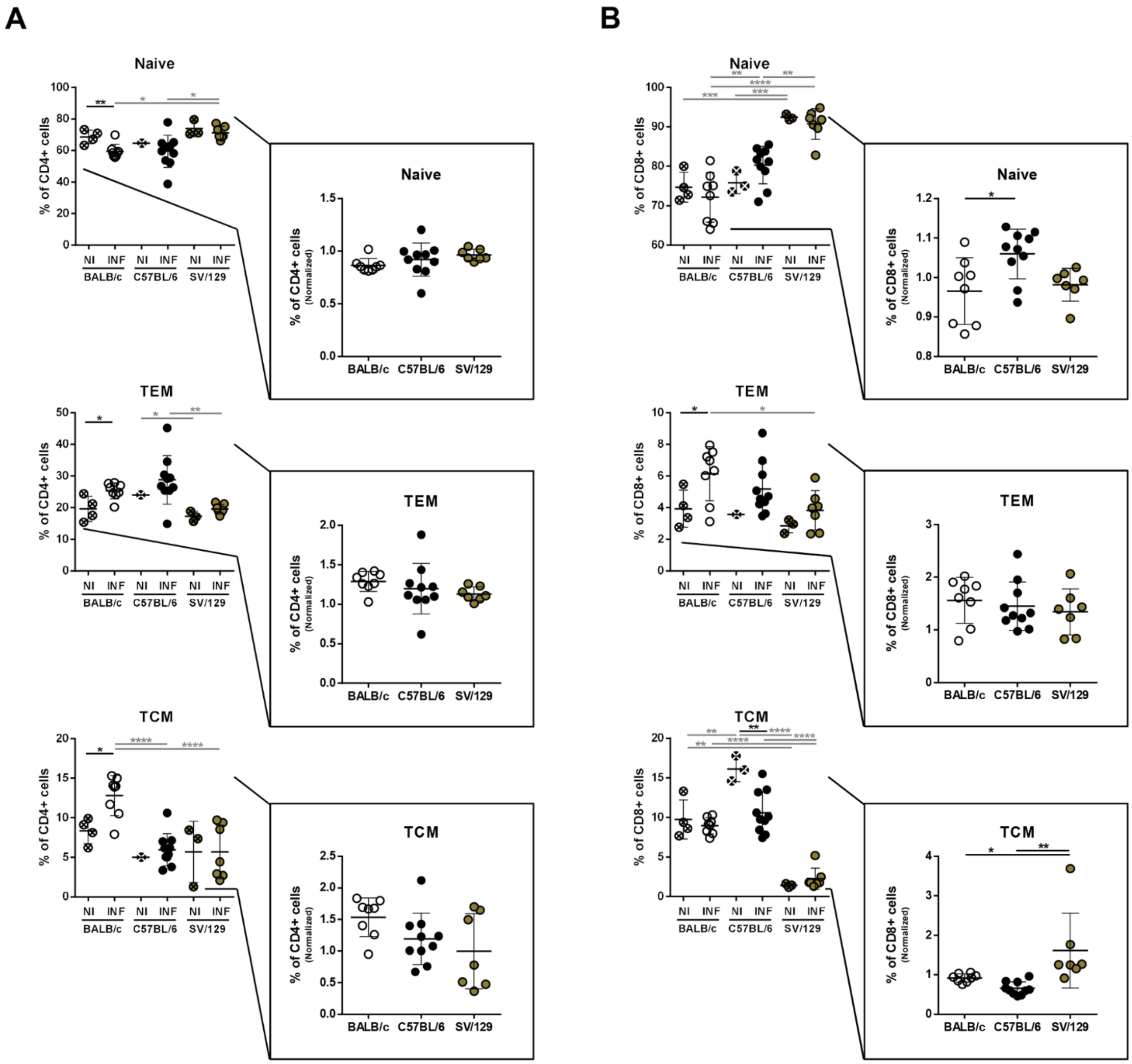

Supplementary figure 4. Splenic T cell memory compartment of the different mice strains and it´s alteration 8 weeks post *L. infantum* infection. BALB/c (white circles), C57BL/6 (black circles) and SV/129 (brown circles) mice were infected intraperitoneally with $1X10^{8}$ *L. infantum* promastigotes and euthanized 8 weeks after. Splenic CD4+ (A) and CD8+ (B) T cell memory compartment was resolved by flow cytometry based on CD44 and CD62L expression. Results, obtained in at least two independent experiments, are represented both in total percentages (infected and control animals) and normalized (infected in relation to control group average values) as a way to highlight the infection-induced alterations. Each dot represents an animal. Average and SD of the values within each group are shown. Statistical differences are properly identified. One Way ANOVA (with Tukey´s *post hoc* analysis) was used for comparisons between the different murine strains (infected or non-infected; grey lines), as well as for comparison of normalized values (black lines); t-tests (black lines) were performed for comparison between infected animals and controls from the same strain: * p≤0.05, ** p≤0.01, *** p≤0.001 and **** p≤0.0001).
